# Report of Robert Battey, M.D., Delegate to the Alabama Medical Association

**Published:** 1873-07

**Authors:** 


### REPORT OF ROBERT BATTEY, M.D., DELEGATE TO THE ALABAMA MEDICAL ASSOCIATION FROM THE GEORGIA MEDICAL ASSOCIATION.

*Mr. President,*—In obedience to a mandate to me issued by the authority of this Association, I presented myself at the meeting of the State Medical Association of Alabama, held in the city of Tuskaloosa on the 25th, 26th, and 27th March ult., in the capacity of a fraternal delegate from this body.

I have the honor to report that I was cordially received and duly honored, as your accredited representative, by the officers and members of the Alabama Association.

Much time was consumed in the discussion and careful scrutiny of a new constitution—a copy of which is herewith presented—offered by Dr. Cochran of Mobile, which had been for three years under consideration. After some unimportant amendments it was adopted by the requisite two-thirds majority, in spite of a well-tempered but vigorous opposition. The courtesy and kindly forbearance which was exhibited during the warm discussion, and the hearty acquiescence and cordiality which followed the voting in of the new law, are worthy of remark as evidencing a laudable spirit of professional harmony.

A resolution was offered inviting your delegate to address the Association upon Normal Ovariotomy. The address was delivered on the morning of the 27th, and listened to with respectful attention. Upon its conclusion Dr. G. A. Ketchum, of Mobile, reported verbally a case of normal ovariotomy in his own practice.

The patient was married and barren. She had amenorrhœa of seven years' duration, with atrophy of the uterus, great nervous perturbation, with convulsions. Her physical and mental state was deplorable; insanity strongly threatened, and life menaced. All remedial means had failed. The successful case in Georgia encouraged hope from normal ovariotomy. A consultation was held with Prof. Gilmore, of the Mobile College, and others; the operation was decided upon, and performed by Dr. Gilmore, early in January, with complete success. The recovery has thus far been most satisfactory. Dr. K. gives the credit of the operation entirely to Dr. Gilmore. A resolution was passed asking a full report of the case from Dr. G. for publication in the proceedings of the Association.

An interesting report was read by Dr. Desprez, of Leighton, upon the use of veratrum in puerperal eclampsia. Many other reports and papers were read by title and ordered into the hands of the Publishing Committee, for want of time to hear them in detail.

An election for officers was held by ballot and without nomination, as provided in the constitution, and with the following result, namely:

*President*—Dr. G. A. Ketchum, Mobile.
*First Vice-President*—Dr. E. A. Semple, Montgomery.
*Second Vice-President*—Dr. Desprez, Leighton.
*Secretary* (five years)—Dr. E. H. Riggs, Selma.
*Treasurer* (five years)—Dr. J. W. Jackson, Montgomery.
*Censor* (five years)—Dr. Jerome Cochran, Mobile.
*Censor* (four years)—Dr. Jas. Guild, Tuskaloosa.
*Censor* (three years)—Dr. A. G. Mabry, Selma.
*Censor* (two years)—Dr. R. F. Michel, Montgomery.
*Censor*—One year—Dr. G. E. Kumpé, Leighton.

The next meeting of the Association was set down for Selma, on the second Tuesday in April, 1874.

On Wednesday evening, the Association was entertained in the Representative Hall of the old capitol building—now the Baptist Female College—by music from the young ladies, and the address from the orator of the day, Dr. Jourdan, of Birmingham, and with credit to all parties.

On Thursday, by invitation of Dr. P. Brice, Superintendent, the body inspected the Alabama Insane Hospital very fully and satisfactorily, under the guidance of Dr. John Little, Jr., Assistant Physician, the illness of Dr. Brice preventing him from officiating; and subsequently partook of a sumptuous "lunch" in the recreation hall of the institution.

The Insane Hospital impressed your delegate most favorably It does honor to the State of Alabama, and especially so to the administrative abilities of Dr. Brice.

While making this inspection, in visiting the steam laundry, the venerable Dr. James Guild, of Tuskaloosa, the nestor of the Alabama profession, most narrowly escaped the loss of his arm, possibly his life, by becoming entangled in the moving machinery. With electric velocity the icy chill ran to every heart; for the moment respiration ceased, and all keenly felt the anxious suspense, when the stout hearts and strong arms of his brethren came to the rescue, and snatched him from his perilous position.

Attached to the Insane Hospital is a small printing office, from which is regularly issued a little periodical styled *The Meteor*, a copy of which is herewith presented. This periodical is both edited and printed solely by a patient of the Hospital—a gentleman of decided talents, an unfortunate member of our own profession.

After leaving the Insane Hospital, the Association repaired to the neighboring grounds of the University, where they were received by President Lupton, and witnessed a review of the pupils in the institution, under the command of General Johnson. The young men presented a fine appearance, and acquitted themselves handsomely in the evolutions and drill to which they were subjected.

On Thursday evening the members of the Association were pleasantly entertained in the hall of the Methodist Female College, with concert and calisthenic exercises by the pupils of the college, under the direction of President Larabee.

In closing this report, your delegate can not forego a passing tribute to the charming old town of Tuskaloosa, with its broad and handsome avenues flanked and bisected by long lines of noble water-oaks—with its stately and luxurious mansions of the olden time. Here one breathes an atmosphere of refinement and culture quite refreshing to the denizen of one of our busy centres of trade and manufactures; and a tribute, too, to the hospitality of her citizens who entertained the members of the Association in their own families, and lavished upon them every courtesy.

